# How Much Child Sexual Abuse is “Below the Surface,” and Can We Help Adults Identify it Early?

**DOI:** 10.3389/fpsyt.2013.00058

**Published:** 2013-07-15

**Authors:** Erin K. Martin, Peter H. Silverstone

**Affiliations:** ^1^Department of Psychiatry, University of Alberta, Edmonton, AB, Canada; ^2^Department of Neuroscience, University of Alberta, Edmonton, AB, Canada

**Keywords:** child, sexual, abuse, incidence, prevalence

## Abstract

Child sexual abuse (CSA) occurs frequently in society to children aged between 2 and 17. It is significantly more common in girls than boys, with the peak age for CSA occurring when girls are aged 13–17. Many children experience multiple episodes of CSA, as well as having high rates of other victimizations (such as physical assaults). One of the problems for current research in CSA is different definitions of what this means, and no recent review has clearly differentiated more severe forms of CSA, and how commonly this is disclosed. In general we suggest there are four types of behavior that should be included as CSA, namely (1) non-contact, (2) genital touching, (3) attempted vaginal and anal penetrative acts, and (4) vaginal and anal penetrative acts. Evidence suggests that CSA involving types (2), (3), and (4) is more likely to have significant long-term outcomes, and thus can be considered has having higher-impact. From the research to date approximately 15% of girls aged 2–17 experience higher-impact CSA (with most studies suggesting that between 12 and 18% of girls experience higher-impact CSA). Approximately 6% of boys experience higher-impact CSA (with most studies suggesting that between 5 and 8% experience higher-impact CSA). The data also suggests that in over 95% of cases the CSA is never disclosed to authorities. Thus, CSA is frequent but often not identified, and occurs “below the surface” in the vast majority of higher-impact cases. Helping adults to understand “below the surface” CSA might help them to recognize it early, but there are very few indicators specific to CSA, making this a challenging goal to achieve. Nonetheless, given that CSA frequently occurs with other types of abuse, a training program that focuses on both CSA and other abuse may offer a method to allow both early recognition and prevention by adults in the general population.

## Introduction

Child sexual abuse (CSA) is a serious societal problem. There is an elevated risk of medical, psychological, behavioral, and sexual disorders in adults who were sexually abused as children (Maniglio, 2009). It is difficult to determine how frequently CSA occurs because of notable methodological differences between studies (Gilbert et al., 2009a,b). Research in the field of CSA uses a wide range of methodologies resulting in a large amount of variation between estimated rates. This methodological variation makes drawing conclusions about the rate of CSA an inaccurate process. Review articles tend to focus on one type of methodological approach such as prevalence studies (e.g., Finkelhor, 1994a; Pereda et al., 2009) or nationally based incidence studies (e.g., Public Health Agency of Canada, 2010). Comparing results achieved with differing methodologies may be useful in determining overall rates and trends regarding CSA. This review addresses this gap in literature by considering both major methodological approaches, incidence studies and retrospective prevalence studies. In this review we also examine whether or not there are currently tools that can help adults in the general public population identify CSA early. Such a review is necessary to better understand the occurrence of CSA in North America as informed by the current research literature.

One way of considering this problem is to use the analogy of an iceberg, with the relatively small number of disclosed occurrences being those that are “above the surface” and the much larger majority of occurrences that not disclosed to authorities as being “below the surface” (Sedlak, 1991; World Health Organization, 2004; Sedlak et al., 2010). This phraseology is more useful than traditional “incidence” or “prevalence” terms, since the occurrence reported in studies varies widely depending upon which criteria and which group is being studied.

Research exploring the impact of CSA is broad and a full review is beyond the scope of this article. Nonetheless, there is compelling evidence that the occurrence of sexual abuse leads to increased rates of multiple psychiatric disorders, including anxiety disorders, depressive disorders, eating disorders, sleep disorders, post-traumatic stress disorders, and suicide attempts (Chen et al., 2010). It is also possible that CSA can lead to long-term changes in neurobiological development that may make such psychiatric conditions more likely (De Bellis et al., 2011). Longitudinal studies have demonstrated that CSA early in life impacts cognitive development, both during the first 8 years of life (Enlow et al., 2012), and as children become adults (Veltman and Browne, 2001). Furthermore, a history of rape, specifically, further increases the risk of major depression, eating disorders, and post-traumatic stress disorders (Chen et al., 2010), and suggests that certain forms of sexual abuse may have a higher-impact and cause greater rates of long-term negative outcomes. One of the major issues, however, in measuring this is that in current CSA research there are no consistent definitions of the types of abuse that are used across studies (Bolen, 2001; Zwi et al., 2007).

For these reasons, there are four major goals of the current review. The first is to utilize the most recent research using a variety of methodological approaches to determination the likely underlying rates of CSA. These include trying to answer the following questions: what are the rates of CSA; what are the types of CSA; how often do they occur; and is the type of CSA changing with increased internet access? The second goal is to determine how much of the time CSA is reported to authorities, as it is clear that the large majority of children who have experienced CSA are not identified. The third goal is to outline some of the complex research issues that remain, and suggest possible approaches to overcome these. The fourth goal is to provide a preliminary exploration of how adults might be taught to identify CSA early, as well as the extent to which this goal is appropriate.

## Methods

For this review, we searched databases (Medline and PsycINFO) using the following keywords: sexual abuse, prevalence, incidence, rates, occurrence, and epidemiology. We also conducted manual searches of the publications CSA and Journal of CSA. We included articles published from 1990 until December 31, 2012, focusing on studies that included data on North American occurrences. We included incidence studies and prevalence studies when results were generalizable.

## Definition of Child Sexual Abuse

The definition of CSA varies, from those definitions that are inclusive of a wide range of activities to those definitions that are restricted to very few and severe actions (e.g., CSA being defined only if incest occurred) (Pereda et al., 2009). The World Health Organization (2003) provides the following definition:

“Child sexual abuse is the involvement of a child in sexual activity that he or she does not fully comprehend, is unable to give informed consent to, or for which the child is not developmentally prepared and cannot give consent, or that violates the laws or social taboos of society. Child sexual abuse is evidenced by this activity between a child and an adult or another child who by age or development is in a relationship of responsibility, trust or power, the activity being intended to gratify or satisfy the needs of the other person” (p. 75).

## Types of Child Sexual Abuse

Another way to conceptualize CSA is by considering types of occurrence. There is a large range of behaviors reported as being CSA, i.e., involving sexual acts with older people or people in positions of trust, power, and/or authority. Previous research has divided the occurrence of CSA into types (e.g., Anderson et al., 1993; Public Health Agency of Canada, 2010; Sedlak et al., 2010). In keeping with this practice, we propose that one way to consider the wide range of behaviors is to combine them into one of four types of abuse: (1) non-contact (e.g., having somebody expose him/herself to the child or being made to watch an individual masturbate); (2) genital touching (e.g., where an individual touches the genitals of the child with his/her hands or mouth, or where the child is made to touch the genitals of the perpetrator with his/her hands or mouth); (3) attempted vaginal and anal penetrative acts (e.g., an older person attempting to insert an object, finger, or penis into the vagina or anus); (4) vaginal and anal penetrative acts (e.g., the perpetrator inserts an object, finger, or penis into the vagina or anus of the child). A child is any person up to, and including the age of 17 years old. For older children, peer sexual assault (e.g., by those who are only 1–3 years older than the victim) can be conceptualized as separate from CSA because the concept of consent becomes an issue, although there are clearly overlapping issues and negative outcomes frequently occur (Danielson and Holmes, 2004; Howard and Wang, 2005; Sherrill et al., 2011).

There is evidence that experiencing CSA types (2), (3), and (4) has greater long-term consequences than type (1). It is also possible that this may also represent an increase in negative impacts since, for example, the occurrence of rape [CSA type (4)] is more likely to be associated with subsequent psychiatric disorders than other forms (Chen et al., 2010). The level of severity of CSA is also shown to be associated with the level of trauma and somatization in adults (Zink et al., 2009), as well as greater occurrence of adverse sexual health indicators (Lacelle et al., 2012). Additionally, penetrative abuse may be at least moderately correlated with psychological and social problems in women (Briere and Jordon, 2009), and a significantly higher likelihood of having contact with a mental health agency (Cutajar et al., 2010). Penetrative abuse is also associated with elevated risk of alcohol problems, having used illicit drugs, suicide attempts, and marriage to an alcoholic (Dube et al., 2005). More severe abuse (i.e., penetrative abuse and abuse involving multiple offenders) is also associated with more severe problems such as high-risk sexual behaviors (Spring and Friedrich, 1992).

From these findings there appears to be a complex interaction between multiple factors, as well as the severity of abuse, that impacts the development of psychiatric disorders and health problems. The following factors are all associated with increased negative impact of CSA in adulthood: an early age at first abuse episode (Briere and Jordon, 2009; Zink et al., 2009; Liu et al., 2012), the number of abusive episodes (Felliti et al., 1998), longer duration of abuse (Beitchman et al., 1992), the presence of coercion during abuse (Dube et al., 2005; Zink et al., 2009), use of force or threat of force (Beitchman et al., 1992), more than one perpetrator (Cutajar et al., 2010), parental mental illness, criminal activity, and substance use (Bailey and McCloskey, 2005), level of marginalization (Briere and Jordon, 2009), and abuse perpetrated by a father or father figure (Beitchman et al., 1992). The interaction between factors is complex and not yet well understood (Briere and Jordon, 2009), although dysfunction in the family of origin (i.e., early parental separation, family violence, and lack of parental warmth) (Weiss et al., 1999) and the occurrence of other types of abuse are shown to influence the later impact of CSA (Briere et al., 2008). There may be protective factors such as resiliency, blame placed on the offender rather than child, social support, and early intervention that mitigate some of the effects (Yancey et al., 2011). Nonetheless, the evidence supports suggestions that the more severe forms of abuse, i.e., what we have termed types (2), (3), and (4), are associated with more negative long-term outcomes. For this reason we suggest that these types of CSA may best be referred to as “higher-impact” CSA.

## Frequency of Childhood Sexual Abuse

Accurate estimation of the frequency of CSA is important because it informs assessment, intervention, treatment, funding, and policy-making decisions. However, accurately and reliably estimating its occurrence is difficult (Gilbert et al., 2009a), even though researchers consistently regard the high frequency as serious (Anderson et al., 1993; Finkelhor, 1994a,b; Sedlak, 2001; Finkelhor et al., 2005; Pereda et al., 2009).

**Figure 1:**
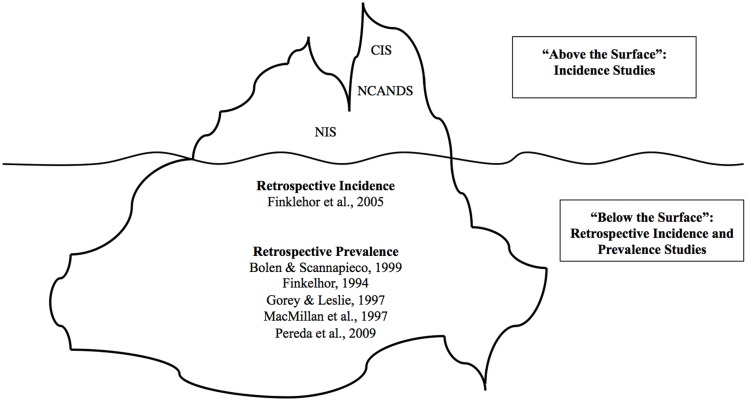
**Iceberg analogy of CSA research studies**. This figure shows summary data for “above the surface” and “below the surface” childhood sexual abuse (CSA). Incidence rates for both the United States in the National Incidence Studies (NIS) and for the Canadian Incidence Studies (CIS), with the latter only reporting on verified cases reported to child welfare services. There is no incidence rate for NCANDS because it reports a percentage of total investigations rather than an estimated rate of occurrence.

There are generally two ways used to estimate the rate of occurrence of CSA, namely *incidence* studies and *retrospective prevalence* studies. Incidence studies measure the number of new cases occurring during a 1-year period while prevalence studies estimate the number of children sexually abused in childhood (Fallon et al., 2010). Incidence studies utilize official data collected by police, child protective services, and other agencies that serve children, and provide estimates of the occurrence of CSA in a 1-year period (e.g., Sarafino, 1979; U.S. Department of Health and Human Services, 1981; Sedlak and Broadhurst, 1996; Trocmé et al., 2001; Sedlak et al., 2010). Self-report retrospective prevalence studies capture information on abuse that in most cases is not reported to official sources. However, the large difference between incidence studies and retrospective prevalence studies is, in large part, explained by differences in “above the surface” and “below the surface” CSA (Figure 1). A single study (Finkelhor et al., 2005) used a third approach that we refer to as a *retrospective incidence* study by asking youth to report CSA experiences that occurred within the past year. In some ways, this approach may offer a leading method to address issues with current data reports.

## “Above the Surface” CSA

The term “above the surface” CSA is consistent with suggestions from the first NIS of CSA known to official sources (U.S. Department of Health and Human Services, 1981). “Above the surface” sources provide important information about disclosed and/or reported CSA. Data on “above the surface” CSA comes largely from the Canadian Incidence Studies (CIS), the NIS, National Child Abuse and Neglect Data System (NCANDS). The NIS and CIS are nationally representative studies estimating American and Canadian incidence of CSA respectively. Fallon et al. (2010) provides a comprehensive review of the details of each of these studies. The reported rates of CSA by each of the studies are presented in Table 1.

**Table 1:** **North American rates of reported child sexual abuse (CSA) from national incidence studies**.

Incidence study	County	CSA rate
NIS-2, 1991	United States	2.1/1,000
NIS-3, 1996	United States	4.5/1,000
NIS-4, 2010	United States	2.4/1,000
CIS-1998	Canada	0.89 Substantiated cases/1,000 children
CIS-2003	Canada	0.62 Substantiated cases/1,000 children
CIS-2008	Canada	0.43 Substantiated cases/1,000 children

*This table shows summary data for “above the surface” childhood sexual abuse (CSA) incidence rates for both the United States in the National Incidence Studies (NIS) and for the Canadian Incidence Studies (CIS), with the latter only reporting on verified cases reported to child welfare services*.

The CIS represent the strictest version of the “above the surface” information, providing data from only the very tip of the iceberg. Thus, the CIS incidence estimates we discuss include only CSA that was investigated and then substantiated by child welfare services. CSA occurrences that are not reported to child welfare services, that do not meet strict validation thresholds, or that are investigated by police alone, are not captured in the CIS incidence rates. There have been three nationally representative CIS studies (Trocmé et al., 2001, 2005; Public Health Agency of Canada, 2010). The most recent Canadian NIS reported a substantiated CSA rate of 0.43/1,000 Canadian children (Public Health Agency of Canada, 2010). Note that this represents only 10–20% of the rates in the NIS studies and less than 1% of the rates from other incidence studies in the United States (Finkelhor et al., 2005).

The NCANDS, also includes only CSA that was reported to child welfare agencies. Data is collected and investigated by a child welfare worker in each state in the United States (U.S. Department of Health and Human Services, 2009). Any investigation on child maltreatment or suspected CSA is then reported to NCANDS using their standardized format. NCANDS then is able to report on data from all states about child maltreatment. In 2009, NCANDS reported that 7.3% of the child welfare cases involved sexual abuse. However, they do not give a standardized rate per 1,000 children.

The NIS studies differs from the CIS and NCANDS. The NIS-2, NIS-3, and NIS-4 collected data on maltreated children from child protective services as well as identified professionals, or “sentinels” (Sedlak, 1991; Sedlak and Broadhurst, 1996; Sedlak et al., 2010). The sentinel design expands the reach of the study by capturing CSA that was disclosed to a non-child welfare professional (e.g., to other professionals such as psychologists). Details of each maltreatment case are reviewed to avoid duplication (Sedlak, 2001). The basic design collects data to provide information on relationships between the characteristics of the abused children, the characteristics of the families, and details of their abuse including characteristics of the perpetrator(s) (Sedlak, 2001). The most recent NIS reported the estimate of “above the surface” CSA at 2.4/1,000 American children. Of the “above the surface” studies, the NIS is able to capture the widest range of CSA.

The CIS, NCANDS, and NIS approaches to studying occurrence of CSA have well recognized strengths and weaknesses. Both the NIS and CIS have excellent reliability in regards to which cases are included (Fallon et al., 2010), and the NIS “sentinel” design allows for more abused children to be identified than with child protection services data alone. Researchers improved the study design in NIS-2 when they started using probability proportional to size sampling (Sedlak, 2001). The basic design of subsequent studies for the NIS and CIS allow for comparisons between findings from more recent studies with findings from older studies. They also provide critical insight into the characteristics and features of “above the surface” CSA. Nonetheless, there are very significant limitations associated with these studies, and one recent review concluded that the definitions of CSA used in both the NIS and CIS are limiting and allow for identification of only certain types of CSA (Fallon et al., 2010). Furthermore, individual child welfare workers decide which cases meet the CIS and NCANDS definitions of child maltreatment (Fallon et al., 2010). Most importantly, the NIS, CIS, and NCANDS approach cannot provide information on the experience of children whose abuse is “below the surface.” For all these reasons, the CIS, NCANDS, and NIS are unlikely to accurately represent the true occurrence of CSA.

## “Below the Surface” CSA

Several studies have examined the rates of “below the surface” CSA by exploring the number of adults in a sample population who report that they experienced CSA (e.g., Anderson et al., 1993; Finkelhor, 1994a; MacMillan et al., 1997; Bolen and Scannapieco, 1999). The CSA experiences identified by these studies include “above the surface” CSA occurrences (i.e., CSA that was reported to an official source), abuse that was not disclosed to an official source but was disclosed to another person such as a family member, and abuse that was not disclosed in childhood. One review reported that studies have found prevalence rates for women as low as 2% and as high as 45% (Bolen and Scannapieco, 1999) while another review reported studies citing rates for CSA in women ranging from 7 to 36% (Finkelhor, 1994a). Taking all available information, one review concluded that the likely “true” rates of CSA occurring throughout childhood for women was between 20 and 30% and for men just below 10% (Pereda et al., 2009). Another review, after controlling for some of the factors responsible for variance (e.g., number of screening questions, sample size, and date of study) concluded that the rate of CSA for women was likely to be between 30 and 40%, and for men between 3 and 13% (Bolen and Scannapieco, 1999). A review focusing on North American data concluded that CSA prevalence rates were 15% for women and 7% for men (excluding the broadest, non-contact, categories), but noted the need for a much better conducted incidence study (Gorey and Leslie, 1997). Since this date, studies have also looked at the rates of CSA in youth who are lesbian, gay, or bisexual, and (based upon data from the 1990s) concluded that there were higher rates of CSA in this group (Saewyc et al., 2006).

## Retrospective Incidence Study

Adult retrospective studies ask adults to report what occurred when they were children, creating a potential issue with the data. Because the abuse occurred during childhood, with sample age ranges from 18 to 65 years old, some adults are reporting CSA that occurred decades earlier. This creates two problems. First, the data collected is potentially flawed because CSA sometimes occurred many years earlier. Second, these studies do not necessarily give an accurate picture of what children and youth are currently experiencing in regards to CSA. One study (Finkelhor et al., 2005) that we refer to as a retrospective incidence study has done well to overcome these concerns.

To date, there is only a single retrospective incidence study that avoids many of the problems previously identified (Finkelhor et al., 2005). This study asked over 2,000 children about their experiences of victimization in the past year (an immediate caregiver answered questions for children up to age 9) providing insight into what children and youth have recently experienced, and may be more accurate in recording (compared to asking adults the same questions). This study found that 9.6% of the girls and 6.7% of the boys interviewed in their sample reported some kind of CSA in the past year, and in general girls experienced more CSA than boys. Of these, a total of 4.2% of girls reported some form of sexual assault in the previous year compared to 2.2% of boys. Most sexual assaults and rapes reported occurred in the age range 13–17, when 6.7% in this age range experienced a sexual assault in the previous year. Importantly, most children were likely to have experienced additional types of victimization during the same year, with 82% also being physically assaulted, 84% witnessing or experiencing indirect victimization, and 70% experiencing property victimization (Finkelhor et al., 2005). These individuals were also likely to experience more than one episode of sexual assault, although the degree to which this occurred was not specifically reported. It should also be noted that in this study nearly 6% of youth (or their parents on their behalf) refused to answer questions about sexual victimization, so it is possible that the rates reported in this study underestimate the actual incidence of CSA.

## What is Most Accurate Estimate for CSA?

Taking all the “below the surface” data together, the findings from studies to date suggests that approximately 4% of girls and 2% of boys experience childhood sexual assault each year, with the majority occurring in the teenage years. Since it is likely that these are often individuals who are re-victimized, it is likely that these numbers are not cumulative, i.e., that there is not an additional new cohort of 4% of girls experiencing CSA each year, and other data do not support such a suggestion either. These rates of reported CSA may also reflect the increase in CSA with age.

It can be clearly seen that the differences in the rate of CSA reported by different studies is dramatic. For example, the annual incidence of all types of CSA identified by United States sentinels (child welfare workers and other identified professionals) was in the range of 2.1–4.5/1,000 children (Sedlak, 1991; Sedlak and Broadhurst, 1996; Sedlak et al., 2010) compared to an incidence rate for any form of sexual victimization of 82/1,000 in a large well-controlled retrospective 1-year incidence study in the United States (Finkelhor et al., 2005). These findings imply that the “above the line” estimates identify only 3–5% of actual cases, which suggests that between 95 and 97% of CSA occurrences are “below the surface.” Put another way, it is likely that at least 95% of CSA is not reported to authorities. Thus, estimates of the rate of CSA varies largely depending upon the source of information and consideration of other relevant factors, such as the definition of CSA, the methodology used, and the sample/sampling procedures, with all these factors contributing to the varying estimates of the incidence and prevalence rates for CSA (Anderson et al., 1993; Leventhal, 1998).

Taking all of the published information to date, we believe the data suggests that approximately 15% of girls experience higher-impact types of CSA (i.e., excluding non-contact experiences), with a likely range of between 12 and 18%. Most of this occurs during the teenage years (i.e., from 13 to 17). However, it should be recognized that given the significant limitations in the data (see below for further discussion of this), that this conclusion must remain a “best guess.”

It is also important to note that many CSA victims will be subject to repeated CSA, and that most victims will also experience other types of non-sexual abuse and victimization. Both of these are likely to be important factors in determining longer-term impacts on the individual. For boys the rates are lower, and the likely rates of higher-impact CSA are around 6%, with the range being from 5 to 8% (again, recognizing that these numbers are a “best guess”). These rates are for those aged 2–17 in North America, but it is possible that these rates vary significantly in other countries. These estimates are very high when compared with the strict and substantiated CSA estimates provided from reported “above the surface” data, with the most conservative data (i.e., that which requires confirmation) suggesting incidence rates of only 0.3% for Type 4 CSA (involving vaginal or anal penetration) (CIS 2001). This large difference between the “above the surface” and “below the surface” data supports suggestions that over 95% of CSA is never reported to authorities. This is very concerning for those who focus on intervention to prevent CSA and treatment of CSA (Lyon and Ahern, 2011).

## Ongoing Limitations in Understanding the Rates for CSA

Despite study findings to date, it is also very important to recognize the significant limitations of current research. While CSA rates by type are reported in many studies (U.S. Department of Health and Human Services, 1981; Sedlak, 1991; Trocmé et al., 2001; Finkelhor et al., 2005), there is little published research on the type of sexual abuse according to age and gender. To date, there is only one study that reports CSA occurrence by type, age group, and gender (Finkelhor et al., 2005). Other studies separate age as percentage of the total (Trocmé et al., 2001, 2005) and by gender (Trocmé et al., 2001). It is clear that more research is needed to determine relationships between age group, gender, and type of CSA.

## Rates by Gender

The data is highly consistent in finding that that girls experience CSA at a higher rate than boys, with the relative rates being between 1.5 and 5.5 times as frequently (Sedlak, 1991; Finkelhor, 1994a; Fergusson et al., 1996; Sedlak and Broadhurst, 1996; MacMillan et al., 1997; Bolen and Scannapieco, 1999; Finkelhor et al., 2005; Pereda et al., 2009; Sedlak et al., 2010). The data from several studies including the United States. NIS suggest that the degree of gender disparity changes with age (U.S. Department of Health and Human Services, 1981; Sedlak, 1991; Sedlak and Broadhurst, 1996). Studies that consider CSA rates by age and gender find that the rates for younger children (age 0–7) are similar for girls and boys, but that the rate in older children are significantly higher for girls (Trocmé et al., 2001, 2005; Finkelhor et al., 2005). The disparity in rates by gender increases with age, even when removing peer assault and considering only abuse perpetrated by adults. Thus, data suggests that at younger ages, girls and boys experience CSA at similar rates, but as girls get older they experience CSA at increasing rates. In contrast, boys have the opposite trend. At younger ages the rate is similar to girls but as age increases the rate of CSA decreases in boys. This disparity does not affect the overall finding that girls experience more CSA than boys. This aspect of CSA has not been emphasized in previous findings, and more research is needed to confirm these suggestions as it may have implications for intervention to identify or prevent CSA.

## Rates by Type of CSA

Estimating the rates of specific types of CSA is even more difficult that estimating the overall occurrence of CSA because few studies separate occurrence of CSA by into types of CSA (Table 2). Despite the difference in methodology, findings from the CIS-2, and Finkelhor et al.’s (2005) study suggest that CSA involving genital contact occurs more frequently than the other types of CSA (Trocmé et al., 2001). When reported, CSA involving genital contact occurred at rates between four and eight times more frequently than penetrative abuse (Trocmé et al., 2001; Finkelhor et al., 2005). The NIS-2 data however, reports only a slightly higher rate of genital contact abuse (0.9/1,000) than penetrative abuse (0.8/1,000). The other types (penetration attempted, penetration completed, and exposure) occur at similar rates to each other (Trocmé et al., 2001; Finkelhor et al., 2005). The NIS-2 data reflects a similar lower rate for the remaining types of CSA. Thus, from the evidence to date there are not consistent findings regarding the type of CSA, and more research is clearly needed on this. Simply identifying whether CSA was type (1), (2), (3), or (4) would allow much better comparisons from research findings between different studies.

**Table 2:** **Rates for incidence of child sexual abuse (CSA) by type of abuse**.

Study	Penetration	Genital contact	Exposure of genitals to child	Attempted penetration	Exploitation: prostitution or pornography
TYPE OF ABUSE					
NIS-1	0.3/1,000	0.2/1,000	0.1/1,000
NIS-2	0.8/1,000	0.9/1,000	0.5/1,000
CIS-1998	0.29/1,000	0.82/1,000	0.21/1,000	0.29/1,000	0.1/1,000
Finkelhor et al. (2005)	4/1,000	32/1,000	4/1,000	18/1,000	

*This table shows summary data for childhood sexual abuse (CSA) by type (where available) for incidence studies in both the United States National Incidence Studies (NIS) and the Canadian Incidence Studies (CIS), and compares these “above the surface” studies to an incidence study that includes “below the surface” rates (Finkelhor et al., 2005). Note that some studies have reported rates for several types of abuse together and the Finkelhor study did not examine rates for exploitation*.

## Are There Changes in the Rates of CSA?

Variations in research methods make it difficult to assess whether or not the incidence of CSA is changing. Understanding changes in incidence of CSA is critical because it will inform prevention, intervention, and treatment approaches (Collin-Vézina et al., 2010). For example, CSA prevention programs that target children became increasingly common in the 1980s and 1990s. If these programs worked as prevention tools, then rates of CSA should have decreased (Leventhal, 1998). If no such decrease is evident, then several explanations are possible. These programs may not be working at reducing the occurrence of CSA, there may be other variables accounting for this lack of efficacy, and/or better reporting process that may be a factor in increased reporting rates of CSA seen in North American (Public Health Agency of Canada, 2010; Sedlak et al., 2010). It is not clear however, if reporting rates are increasing consistently as adolescent reporting rates of CSA did not change significantly between 2003 and 2008 (Finkelhor et al., 2010a,b). Nonetheless, in order to determine if the rate of CSA has been changing, methodological issues in the research must first be addressed (Fallon et al., 2010).

Some of the variation between studies can be attributed to methodological issues involving variations in CSA definitions, differences in screening questions used, type of data collection, and sample size and type. The definition of CSA impacts results. Studies with broad definitions yield higher rates than those with narrow ones (Finkelhor, 1994b; Pereda et al., 2009). Studies that examine only one type of abuse may result in under-estimates of the occurrence of CSA because each type, in isolation, is inadequate (Shaffer et al., 2008) because it missed the overall occurrence of CSA. For women, higher number of screening questions predicts higher prevalence rates (Anderson et al., 1993; Bolen and Scannapieco, 1999). More descriptive and detailed questions are shown to result in higher rates being reported in reviews of epidemiological studies (Finkelhor, 1994a; Pereda et al., 2009), college samples (Fricker et al., 2003), and community samples (Martin et al., 1993).

The way in which CSA occurrence is determined also impacts resulting prevalence rates. For women, face-to-face interviews may provide opportunity for more detailed screening questions resulting in more disclosure of CSA and higher general prevalence rates but familial CSA may be reported more often in anonymous postal questionnaires (Martin et al., 1993). Differences in sample size and type also account for variation. Smaller sample size predicts lower prevalence rates (Finkelhor, 1994a; Bolen and Scannapieco, 1999). Some studies propose that sample type (national, state, clinical, or community) is a factor in prevalence variation but in their corrective meta analysis, Bolen and Scannapieco (1999) found that type (national, state, or community) did not result in statistically significant differences in prevalence. Clinical samples tend to be small and non-generalizable (MacMillan et al., 1997).

Perhaps because of these problems in methodology, previous reviewers have differed about whether rates are changing. In 1997, one review suggested that rates have had not changed in the previous 30 years (Gorey and Leslie, 1997). More recent reviews suggest that rates are decreasing, particularly in substantiated rates in American studies (Finkelhor et al., 2010a,b; Sedlak et al., 2010). In contrast, a recent Canadian study concludes that it is too soon to determine that rates of CSA are decreasing (Collin-Vézina et al., 2010), and as we have previously noted, that there are so many significant problems with so much of the data that is simply not possible to confidently determine if there have been any changes in incidence or prevalence rates of CSA over the past few decades.

## Possible Role of the Internet

Another important issue to consider in terms of possible changes in the rates of CSA is the potential impact of Internet pornography, particularly child-focused pornography (Wolak et al., 2010). Despite the large amount of publicity given to Internet child pornography (Wolak et al., 2010) it is uncertain if this has altered the occurrence of CSA. It certainly appears to be the case that many individuals charged with offenses based upon viewing child pornography had not previously been identified as sexual offenders against children (Briggs et al., 2011; Nielssen et al., 2011). This group also tends to have different characteristics to previous CSA offenders (Niveau, 2010). Thus, it remains unclear about the link between those who view Internet child pornography and those who commit CSA. It has been suggested that up to 32% of individuals who view child pornography may re-offend (or at least, be charged – more may re-offend without being caught), but a much smaller subset (4%) are actually charged with a new violent offense in the 6 years following their initial conviction (Eke et al., 2011). To date, however, research has not been able to demonstrate that the Internet has increased rates of CSA, although more youth do appear to have experienced on-line sexual harassment (Jones et al., 2012). Nonetheless, it does appear that for high-risk CSA offenders pornography increases the risk of offending (Kingston et al., 2008). More research is clearly needed to determine the effect of easier access to child pornography on rates of CSA.

Other childhood factors are also associated with the occurrence of CSA. Family conflict, lower parental bonding, and parental problems with substance abuse or criminality are also demonstrated to correlate with occurrence of CSA (Fergusson et al., 1996). Given changes in economic opportunity, and changes in substance abuse rates over the past 15 years (Merikangas and McClair, 2012), research determining how changes in these factors may be impacting CSA rates also needs to be carried out before it is clear if rates are changing, and if so, what the cause may be.

## Can We Help Adults to Identify CSA Early?

Given the previously discussed rates of CSA and the potentially serious impacts (Maniglio, 2009) a critical questions remains to be asked, namely can we teach adults in the general population to identify the occurrence of CSA early? Early identification could mean early intervention and treatment, potentially mitigating some of the longer-term impact. An extensive body of research already exists on identifying CSA in forensic settings (see Faust et al., 2009) and medical settings (Adams, 2001; Heger et al., 2002; Horner, 2009; Adams et al., 2012). However, there is a dearth of research exploring the effectiveness and appropriateness of teaching adults in the general population to identify CSA.

An additional question is how effective is it be to teach adults about the indicators of CSA? Evaluated adult-targeted CSA education approaches are shown to be effective at increasing participant’s knowledge about CSA in terms of self-reported knowledge gains (Self-Brown et al., 2008; Kenny, 2009), when assessed by comparing pre-test results to post-test results (Rheingold et al., 2007; Bowman et al., 2010), and when comparing participants to non-participants (Hébert et al., 2002; Rheingold et al., 2012). Teaching adults about CSA is likely to increase their knowledge about the selected teaching topics. If programs were to teach adults about early identification of CSA, likely adults would increase their knowledge in this area. Despite this, there is no evidence that such programs actually alter detection rates for CSA or that they increase prevention of CSA.

Given this, it is uncertain how appropriate is it to teach adults to identify CSA. There are very few definitive indicators of CSA and many childhood behaviors that might first appear concerning, such as sleep disturbances, are actually quite common in all children (Faust et al., 2009). Furthermore, a reproducible and reliable form of assessment for determining whether or not a child was sexually abused has yet to be developed for professionals (Faust et al., 2009), making it appear unlikely that we can reliably teach adults in the general population to accurately identify CSA.

Nonetheless, because psychological and behavioral indicators of CSA are so non-specific (i.e., they also occur after a child experiences non-CSA stressors, abuse, and neglect) it may not be appropriate to teach adults to use psychological and behavioral indicators to identify CSA alone. Therefore, adults in the general public could be trained to identify more general behaviors indicative of stress, and to then ask these children about CSA. It is likely that adults can be trained to recognize generally concerning psychological and behavioral occurrences in children and to respond appropriately. While these occurrences may not necessarily indicate CSA specifically, it remains in the best interest of the child for adults to respond with care and concern, as it is possible some type of abuse and neglect could be occurring. This is particularly relevant since studies indicate that many children who experience CSA also experience other types of abuse and neglect (Felliti et al., 1998; Finkelhor et al., 2005, 2007). Qualitative studies exploring children’s disclosure experiences (Heger et al., 2002; Alaggia, 2004, 2010; Staller and Nelson-Gardell, 2005; London et al., 2008) can also be used to inform program content aimed to increase adult’s accurate awareness of CSA disclosure and appropriate responses to disclosures.

Based on our current knowledge, it may be most appropriate to teach adults, more generally, about signs that might indicate that a stressor occurring in a child’s life. This approach is appropriate for two main reasons: firstly, it allows for the opportunity of intervention and treatment of a range of types of abuse and neglect, recognizing the often overlapping nature of types of abuse and neglect; and secondly, it acknowledges our current lack of knowledge about what concerning signs indicate CSA specifically. For such a training course, this over-arching framework of teaching indicators of abuse can be done within a CSA specific education program so that information about CSA is also conveyed. This may prove to be a fruitful approach, but will need rigorous research to ensure both appropriate design and measurement of appropriate outcomes.

## Conclusion and Future Directions

Child sexual abuse is a major health and psychological problem with major impacts on individuals and on society. Despite its importance, it is not clear exactly how commonly this occurs, or if the incidence of CSA is increasing, decreasing, or staying the same. There are important gaps in knowledge about the occurrence of CSA as it relates to child age, gender, and type of abuse. The impact of Internet child pornography on CSA rates is also unclear. Nonetheless, findings to date are consistent that most CSA occurs in girls and is not reported to authorities. Preventing and treating CSA should be a major priority for both research and society, as the longer-term effects, particularly of higher-impact CSA are profound and potentially life-long. Future research must consider the methodological issues highlighted in this article in order to provide accurate information about the yet unanswerable questions about CSA in North America.

Additionally, while it may not be ideal to teach adults to recognize indicators of CSA, there is reason to teach adults to recognize generally concerning signs in children that could indicate some type of stressor in the child’s life. There is a clear requirement for education programs for adults who may be able to help both decrease the incidence of CSA (by limiting access and increasing awareness) and to help increase disclosure rates (by being able to help those children who may be trying to disclose). For these reasons there is a clear need to develop specialized CSA education programs that are scientifically based and rigorously evaluated. The goal for all societies should be to completely eradicate CSA, and this will only occur if better educational programs are available and are widely implemented.

## Conflict of Interest Statement

The authors declare that the research was conducted in the absence of any commercial or financial relationships that could be construed as a potential conflict of interest.
